# Cumulative financial stress as a potential risk factor for cancer-related fatigue among prostate cancer survivors

**DOI:** 10.1007/s11764-020-00906-7

**Published:** 2020-08-01

**Authors:** Liya Lu, Anna Gavin, Frances J. Drummond, Linda Sharp

**Affiliations:** 1 Population Health Sciences Institute, Newcastle University Centre for Cancer, Newcastle University, Newcastle, England; 2 Northern Ireland Cancer Registry, Queen’s College Belfast, Belfast, Northern Ireland; 3 CancerResearch@UCC, University College Cork, Cork, Ireland

**Keywords:** Prostate cancer, Financial stress, Cancer-related fatigue, Ireland

## Abstract

**Introduction:**

Cancer-related fatigue (CRF) is the most commonly reported treatment-related side effect of prostate cancer (PCa). Recognition of financial hardship among cancer survivors is growing. We investigated, for the first time, associations between levels of financial stress and CRF among PCa survivors.

**Methods:**

We used data from PCa survivors who had been identified through two population-based cancer registries covering the Republic of Ireland and Northern Ireland and had completed a postal questionnaire. CRF was measured by the fatigue subscale of the EORTC QLQ-C30. Financial stress was assessed as household ability to make ends meet (i) pre-diagnosis and (ii) at questionnaire completion (post-diagnosis). Multivariable logistic regression was used to relate financial stress to clinically important CRF (fatigue subscale score ≥ 39 of a possible 100).

**Results:**

Two thousand four hundred fifty-eight PCa survivors were included. Of these, 268 (10.9%) reported pre-diagnosis financial stress only, 317 (12.9%) post-diagnosis stress only and 270 (11.0%) both pre- and post-diagnosis stress (cumulative stress); 470 (19.1%) reported clinically important CRF. After controlling for confounders, survivors with cumulative financial stress exposure were significantly more likely to have CRF (OR = 4.58, 95% CI 3.30–6.35, *p* < 0.001), compared with those without financial stress. There was a suggestion of a dose-response relationship (OR = 1.83, 95% CI 1.27–2.65, *p* = 0.001 for pre-diagnosis financial stress only; and OR = 4.11, 95% CI 3.01–5.61, *p* < 0.001 for post-diagnosis financial stress only).

**Conclusions:**

Financial stress may be an independent risk factor for CRF.

**Implications for Cancer Survivors:**

There may be benefits in targeting interventions for reducing CRF towards survivors with financial stress, or developing strategies to reduce financial stress.

**Electronic supplementary material:**

The online version of this article (10.1007/s11764-020-00906-7) contains supplementary material, which is available to authorized users.

## Introduction

An estimated 1.3 million new prostate cancers (PCas) are diagnosed worldwide each year, and it is the most common cancer among men in the WHO regions of Europe, the Americas and Africa [1]. Prevalence is increasing due to early detection and 5-year survival now exceeds 90% in many populations, including Canada, Brazil, Australia, Japan and much of Northern and Western Europe [2]. Consequently, in many countries, more men are living with PCa than any other form of cancer: for example, there are more than 6 million PCa survivors in the USA [3], 250,000 in the UK [4] and 24,000 in the Republic of Ireland (RoI) [5]. These numbers are projected to rise rapidly [6]. Understanding the needs and outcomes of this growing population is therefore important.

Cancer-related fatigue (CRF) is a prolonged, distressing and subjective sense of physical, emotional or cognitive exhaustion associated with cancer or its treatment [7]. It can result from current mainstream treatments for PCa, particularly androgen deprivation therapy (ADT) [8]. Indeed, it is the most commonly reported treatment-related side effect of PCa [9, 10]. In our own work, we found that PCa survivors had worse scores for fatigue than any other general cancer-related symptoms recorded on the EORTC QLQ-C30 [11]. Others have reported that CRF may be found in up to three-quarters of men with PCa [8]. Clinically significant levels of fatigue among PCa survivors have been associated with severe psychological distress [12].

CRF interferes with functional capacity and is not proportional to recent activity nor relieved by rest or sleep [7]. It can impact on survivors’ lives for years after treatment cessation [13]. Despite this, relatively little research has investigated correlates of CRF among survivors. Most studies have been conducted in breast cancer survivors [14–17] or survivors of common cancer types combined [18]. These suggest that, among other factors, economic variables, such as lower household income, may be associated with higher risk of CRF [14].

Recognition of financial hardship among cancer survivors is growing [19–23]. Survivors may be vulnerable to material financial hardship (henceforth financial stress) [24] due to out-of-pocket costs as a result of the cancer diagnosis (e.g. for medical care, supportive medications, travel to appointments) and lost income because of work absence during cancer treatment and rehabilitation [19]. Such financial stress appears particularly common in survivors with low income or financial stress before diagnosis [25–28] and has been associated with poor psychological well-being and worse quality of life (QoL) in a range of cancers [23, 26, 29–31]. These findings have led to a recent call to increase knowledge of the relationship between financial stress and cancer-related symptoms or side effects, particularly those with physical manifestations [32]; better understanding of this could inform development and targeting of interventions among survivors.

The aim of this study, therefore, was to investigate—for the first time—the association between levels of financial stress (pre-diagnosis, post-diagnosis and cumulative) and CRF among PCa survivors.

## Patients and methods

### Design and setting

The Prostate Cancer Treatment-your experience (PiCTure) study was a cross-sectional study of PCa survivors sampled and recruited via two population-based cancer registries on the island of Ireland: the National Cancer Registry Ireland (NCRI) in the Republic of Ireland (RoI) and the Northern Ireland (NI) Cancer Registry (NICR). The RoI has a mixed public-private healthcare system where individuals who use the public system make modest co-payments for public health services and full payments for prescribed medications unless they have a medical card (entitlement to which is based on means and age). Private health insurance plans generally cover hospital care. NI, part of UK, has a primarily public healthcare system, the National Health Service. The PiCTure study was approved by the College of General Practitioners in the RoI and the Office for Research Ethics Committee in NI [33].

### Identification and recruitment of survivors

All men diagnosed with incident primary invasive PCa (ICD10 C61) between 01/01/1995 and 31/03/2010 and alive 31/03/2011 were identified from NCRI and NICR (RoI = 17,304; NI = 5519). As previously described [11], a country and time since diagnosis-stratified random sample of 12,322 survivors (54% of sampling frame comprising approximately equal numbers < 5 and ≥ 5 years post-diagnosis in each jurisdiction) was screened for eligibility by healthcare providers. Eligible survivors had to be alive and aware of PCa diagnosis, living in RoI/NI, English-speaking, and potentially able to complete a questionnaire.

### Data collection and measures

Following health professional screening, a postal survey was sent to 6659 eligible survivors. Non-respondents were sent up to two written reminders at fortnightly intervals. Questionnaire responses were linked to the cancer registry records to obtain date of birth, date of diagnosis (and hence time since diagnosis), clinical stage and Gleason grade. Full details of the questionnaire content are provided elsewhere [33]. It collected demographic (e.g. marital status, education, employment status) and clinical data (e.g. presence of comorbidities at diagnosis of cancer, treatment received following diagnosis). CRF in the week prior to the questionnaire completion was assessed using the fatigue scale (FA) of the European Organization for Research and Treatment of Cancer Quality of Life Core Questionnaire (EORTC QLQ-C30), a validated instrument for capturing health-related quality of life (HRQoL) in cancer patients/survivors [34, 35]. A FA subscale score ≥ 39 out of a possible 100 indicates the presence of clinically important CRF [36]. To assess pre-diagnosis financial stress, participants were asked to report the level of financial stress they had experienced *just before* their cancer diagnosis. The question was derived from previous research and asked about the ability of the survivor’s household to make ends meet immediately before diagnosis [28, 37]. Responses were on a 6-level Likert type scale ranging from “Very difficult” to “Very easy”. Participants were considered to have pre-diagnosis financial stress if they responded “Very difficult/Difficult/Somewhat difficult”. To assess post-diagnosis financial stress, participants were asked about the period of time *since the cancer diagnosis*; the question asked about the impact of the cancer on the ability of the survivor’s household to make ends meet. Responses were on a 7-level Likert type scale (including a central “no change” option). Participants were considered to have post-diagnosis financial stress if they responded “Much more difficult/More difficult/A little more difficult”. We applied a cumulative perspective to investigate whether survivors with higher levels of financial stress exposure were more fatigued. On this basis, cumulative financial stress exposure was defined as exposure to both pre-diagnosis and post-diagnosis financial stress.

For analysis, the study population was categorised as follows: ≤ 59, 60–69, ≥ 70 years of age at diagnosis; married/living with a partner and other; living alone at diagnosis or not; primary, secondary or ≥ tertiary as highest level of education at diagnosis; working (employed/self-employed), not working (retired, unemployed, unable to work due to disability/sickness) and other employment status; any or no comorbidities at diagnosis; early (localised disease: stage I/II and Gleason score 2–7 at diagnosis), late (locally advanced/advanced disease: III/IV and any Gleason score at diagnosis) unknown extent (other combinations of stage and Gleason score, or unknown stage or Gleason score) of disease; and time since diagnosis (2–5, 5–10, ≥ 10 years. A mutually exclusive hierarchical treatment variable was created based on primary treatment(s) ever received, as follows: radical prostatectomy (RP) with or without other treatments; external beam radiotherapy (EBRT) with or without concurrent ADT; brachytherapy (BT) without EBRT or RP; ADT alone without RP, EBRT or BT; active surveillance/watchful-waiting; and other.

### Statistical analyses

The study population comprised questionnaire respondents who had completed the CRF questions and the questions on financial stress. We used *χ*^2^ tests to compare the socio-demographic and clinical characteristics of this group with those respondents who did not complete these questions. The frequencies and percentages of explanatory variables were summarised for the entire study population. We summarised the frequencies of pre-diagnosis, post-diagnosis and cumulative financial stress across the study population and by key socio-demographic and clinical variables. We then used *χ*^2^ tests to compare subgroups of participants by presence and absence of CRF. Tests for trend were conducted across categories of financial stress and for other variables which were ordinal. Multivariable logistic regression analyses were performed to examine the association between financial stress exposure and CRF, adjusting for covariates. The model fitting involved the following steps: (i) all variables which had a *p* ≤ 0.10 in univariable analyses were fitted together in an initial multivariable model; (ii) variables which had a *p* ≤ 0.05 in this initial model were retained and considered the core model; (iii) variables which were not statistically significant in the core model, or univariable analyses, were fitted individually to the core model and significance checked using likelihood ratio test; any with a *p* value of ≤ 0.05 were retained. In developing the model, we excluded survivors with missing information (providing the percentage missing was < 5%) and those whose treatment was classified as “other” (which included chemotherapy only or unknown treatment). Throughout the model fitting process, we took care to avoid multicollinearity. Variables in the final model had a variance inflation factor < 10 and tolerance > 0.1. The final model had adequate fit (goodness-of-fit test: *p* = 0.533) [38].

## Results

### Study sample

Overall, 3348 PCa survivors responded to the questionnaire (response rate = 54%). Of these, 2458 provided complete data on CRF and financial stress and comprised the current study population. When this study population was compared with the respondents excluded because of incomplete data on CRF and financial stress (*n* = 890), the study population were younger at diagnosis, more often from RoI, and more often working at diagnosis; they more often had early disease and less often were on ADT (*χ*^2^ tests: all *p* < 0.001); they more often had completed higher education (*p* for trend < 0.001) and less often had comorbidities (*χ*^2^ test: *p* < 0.001). There was no significant difference in terms of time since diagnosis (*p* = 0.369).

Of the 2458 participants, the majority were from RoI (81.5%), at least 60 years of age at cancer diagnosis (71.1%), married/living with a partner (83.3%) and had completed only school-level education (69.4%). Around half were diagnosed with early disease (52.5%) and were within 5 years of diagnosis at survey (47.8%) (Table 1).

**Table 1:** Study characteristics of prostate cancer survivors included in the analysis. *N* = 2458

	*n* (%)
Financial stress	
No	1603 (65.2)
Pre-diagnosis only	268 (10.9)
Post-diagnosis only	317 (12.9)
Cumulative^a^	270 (11.0)
Age at diagnosis, years	
≤ 59	710 (28.9)
60–69	1199 (48.8)
≥ 70	549 (22.3)
Jurisdiction	
RoI	2002 (81.5)
NI	456 (18.5)
Marital status at diagnosis	
Married/living with a partner	2047 (83.3)
Other	394 (16.0)
Not reported	17 (0.7)
Living alone at diagnosis	
No	2147 (87.4)
Yes	289 (11.8)
Not reported	22 (0.9)
Highest level of education at diagnosis	
Primary	791 (32.2)
Secondary	914 (37.2)
≥ Tertiary	675 (27.4)
Not reported	78 (3.2)
Employment status, immediately before diagnosis	
Working	1322 (53.8)
Not working/other	1039 (42.3)
Not reported	97 (3.9)
Comorbidities at diagnosis	
No	1148 (46.7)
Yes	1310 (53.3)
Extent of disease at diagnosis^b^	
Early	1290 (52.5)
Late	458 (18.6)
Unknown	710 (28.9)
Treatment^c^	
RP	802 (32.6)
EBRT	1219 (49.6)
BT	109 (4.4)
ADT	177 (7.2)
Active surveillance/watchful-waiting	104 (4.2)
Other	47 (1.9)
Time since diagnosis, years	
2–5	1174 (47.8)
5–10	786 (32.0)
≥ 10	498 (20.2)

^a^Experienced both pre-diagnosis and post-diagnosis financial stress

^b^Early (localised disease: stage I/II and Gleason score 2–7 at diagnosis), late (locally advanced/advanced disease: III/IV and any Gleason score at diagnosis), unknown extent (other combinations of stage and Gleason score, or unknown stage or Gleason score)

^c^Primary treatment(s): a hierarchical variable defined as (i) RP at any time following diagnosis (with/without other treatments); (ii) EBRT with/without concurrent ADT; (iii) BT without previous RP or EBRT; (iv) ADT alone without RP, EBRT or BT; (v) active surveillance/watchful-waiting; and (vi) other (which includes chemotherapy (2 participants) and unknown treatment (45 participants))

*RoI* the Republic of Ireland; *NI* Northern Ireland; *RP* radical prostatectomy; *EBRT* external beam radiotherapy; *BT* brachytherapy; *ADT* androgen deprivation therapy

### Financial stress

**Fig. 1:**
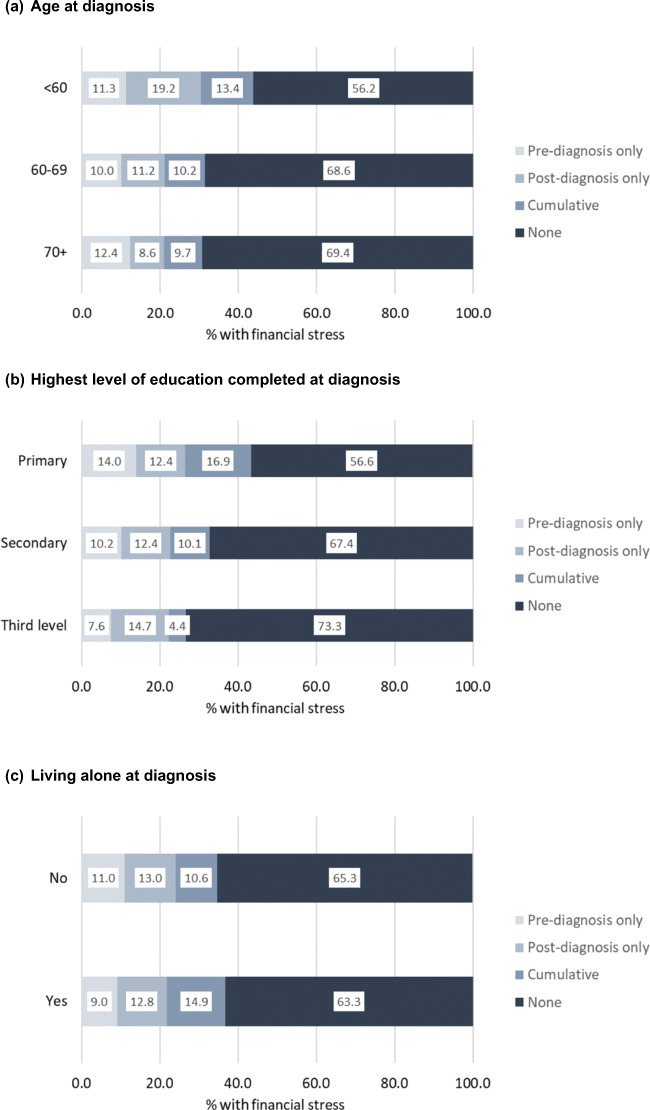

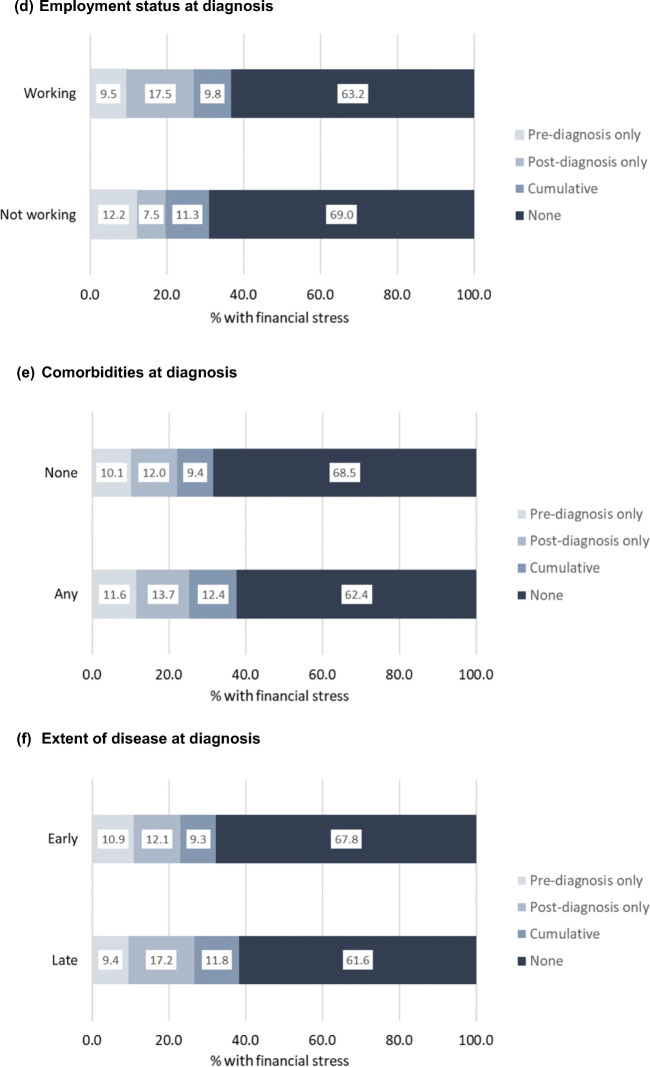
Prevalence of financial stress by key socio-demographic and clinical variables. **a** Age at diagnosis. **b** Highest level of education completed at diagnosis. **c** Living alone at diagnosis. **d** Employment status at diagnosis. **e** Comorbidities at diagnosis. **f** Extent of disease at diagnosis

Overall, 538 (21.9%) reported pre-diagnosis financial stress and 587 (23.9%) reported post-diagnosis financial stress. When these were considered together, 268 (10.9%) reported pre-diagnosis financial stress only, 317 (12.9%) had post-diagnosis financial stress only and 270 (11.0%) reported both (cumulative financial stress); around two thirds had neither pre- nor post-diagnosis financial stress (Table 1). Pre-diagnosis stress only was more common among those with primary education than secondary or tertiary education; those working at diagnosis more often reported post-diagnosis stress than those who were not working; and prevalence of cumulative stress was higher in those < 60, resident in RoI and living alone at diagnosis, with comorbidities, without early disease (Supplementary Table 1; Fig. 1a–f).

### Prevalence and factors associated with clinically important CRF

Of the 2458 participants, 470 (19.1%) scored in the range for clinically important CRF at the time of questionnaire completion. Survivors with financial stress exposure reported clinically important CRF significantly more often compared with those without financial stress; there was a linear trend across categories of financial stress (% reporting CRF: no financial stress, 12.2%; pre-diagnosis financial stress only, 20.5%; post-diagnosis financial stress only, 32.5%; cumulative financial stress, 43.3%; *p* for trend < 0.001) (Table 2). Other variables significantly associated (*χ*^2^ *p* ≤ 0.05) with clinically important CRF were age at diagnosis, jurisdiction, level of education, employment status at time of diagnosis, presence of comorbidities, disease extent and treatment received (Table 2).

**Table 2:** Univariable analysis—associations between financial stress, demographic and clinical variables and cancer-related fatigue: *p* values from *χ*^2^ tests, odds ratios (ORs), 95% confidence intervals (CIs) and *p* values from tests of significance

	Cancer-related fatigue				
	Yes (*n* = 470)	No (*n* = 1988)	*p*^a^	OR (95% CI)	*p*
	*n* (%)	*n* (%)			
Financial stress					
No	195 (12.2)	1408 (87.8)	< 0.001^b^	1.00	–
Pre-diagnosis only	55 (20.5)	213 (79.5)		1.86 (1.34–2.60)	< 0.001
Post-diagnosis only	103 (32.5)	214 (67.5)		3.48 (2.63–4.59)	< 0.001
Cumulative^c^	117 (43.3)	153 (56.7)		5.52 (4.16–7.33)	< 0.001
Age at diagnosis, years					
≤ 59	139 (19.6)	571 (80.4)	0.032^b^	1.00	–
60–69	193 (16.1)	1006 (83.9)		0.79 (0.62–1.00)	0.053
≥ 70	138 (25.1)	411 (74.9)		1.38 (1.06–1.80)	0.018
Jurisdiction					
RoI	364 (18.2)	1638 (81.8)	0.013	1.00	–
NI	106 (23.3)	350 (76.7)		1.36 (1.07–1.74)	0.013
Marital status at diagnosis					
Married/ living with a partner	378 (18.5)	1669 (81.5)	0.057	1.00	–
Other	89 (22.6)	305 (77.4)		1.29 (0.99–1.67)	0.057
Living alone at diagnosis					
No	401 (18.7)	1746 (81.3)	0.092	1.00	–
Yes	66 (22.8)	223 (77.2)		1.29 (0.96–1.73)	0.092
Highest level of education at diagnosis					
Primary	192 (24.3)	599 (75.7)	< 0.001^b^	1.00	–
Secondary	171 (18.7)	743 (81.3)		0.72 (0.57–0.91)	0.005
≥ Tertiary	92 (13.6)	583 (86.4)		0.49 (0.37–0.65)	< 0.001
Employment status, immediately before diagnosis					
Working	208 (15.7)	1114 (84.3)	< 0.001	1.00	–
Not working/other	233 (22.4)	806 (77.6)		1.55 (1.26–1.91)	< 0.001
Comorbidities at diagnosis					
None	157 (13.7)	991 (86.3)	< 0.001	1.00	–
Any	313 (23.9)	997 (76.1)		1.98 (1.60–2.45)	< 0.001
Extent of disease at diagnosis^d^					
Early	210 (16.3)	1080 (83.7)	< 0.001	1.00	–
Late	117 (25.6)	341 (74.4)		1.76 (1.36–2.28)	< 0.001
Unknown	143 (20.1)	567 (79.9)		1.30 (1.02–1.64)	0.030
Treatment^e^					
RP	115 (14.3)	687 (85.7)	< 0.001	1.00	–
EBRT	265 (21.7)	954 (78.3)		1.66 (1.31–2.11)	< 0.001
BT	11 (10.1)	98 (89.9)		0.67 (0.35–1.29)	0.231
ADT	58 (32.8)	119 (67.2)		2.91 (2.01–4.22)	< 0.001
Active surveillance/watchful-waiting	15 (14.4)	89 (85.6)		1.01 (0.56–1.80)	0.982
Time since diagnosis, years					
2–5	233 (19.9)	941 (80.1)	0.061	1.00	–
5–10	130 (16.5)	656 (83.5)		0.80 (0.63–1.01)	0.065
≥ 10	107 (21.5)	391 (78.5)		1.11 (0.85–1.43)	0.446

^a^*χ*^2^ test; unknown group is excluded if < 5% has missing data; other treatment group is excluded

^b^*p* for trend

^c^Experienced both pre-diagnosis and post-diagnosis financial stress

^d^Early (localised disease: stage I/II and Gleason score 2–7 at diagnosis), late (locally advanced/advanced disease: III/IV and any Gleason score at diagnosis), unknown extent (other combinations of stage and Gleason score, or unknown stage or Gleason score)

^e^Hierarchical variable based on primary treatment(s) reported

*RoI* the Republic of Ireland; *NI* Northern Ireland; *RP* radical prostatectomy; *EBRT* external beam radiotherapy; *BT* brachytherapy; *ADT* androgen deprivation therapy

After controlling for significant demographic and clinical confounders in the multivariable model, the association between financial stress exposure and CRF persisted. There was suggestion of a dose-response relationship whereby the risk of CRF increased with increasing financial stress (OR = 1.83, 95% CI 1.27–2.65, *p* = 0.001 for pre-diagnosis financial stress only; OR = 4.11, 95% CI 3.01–5.61, *p* < 0.001 for post-diagnosis financial stress only; and OR = 4.58, 95% CI 3.30–6.35, *p* < 0.001 for cumulative financial stress) (Table 3).

**Table 3:** Multivariable logistic regression analysis—financial stress and demographic and clinical variables significantly associated with cancer-related fatigue: odds ratios (ORs) with 95% confidence intervals (CIs) and *p* values

	Multivariable analyses^a^		*p*^b^
	OR (95% CI)	*p*	
Financial stress			< 0.001
No	1.00	–	
Pre-diagnosis only	1.83 (1.27–2.65)	0.001	
Post-diagnosis only	4.11 (3.01–5.61)	< 0.001	
Cumulative^c^	4.58 (3.30–6.35)	< 0.001	
Age at diagnosis, years			0.007
≤ 59	1.00	–	
60–69	0.68 (0.51–0.92)	0.011	
≥ 70	0.98 (0.67–1.44)	0.918	
Jurisdiction			0.020
RoI	1.00	–	
NI	1.45 (1.07–1.96)	0.016	
Highest level of education at diagnosis			0.006
Primary	1.00	–	
Secondary	0.97 (0.75–1.27)	0.836	
≥ Tertiary	0.63 (0.46–0.86)	0.004	
Employment status, immediately before diagnosis			0.001
Working	1.00	–	
Not working/other	1.62 (1.23–2.12)	< 0.001	
Comorbidities at diagnosis			< 0.001
None	1.00	–	
Any	1.76 (1.39–2.24)	< 0.001	
Extent of disease at diagnosis^d^			0.005
Early	1.00	–	
Late	1.61 (1.20–2.16)	0.002	
Unknown	1.03 (0.78–1.36)	0.829	
Treatment^e^			0.001
RP	1.00	–	
EBRT	1.57 (1.17–2.09)	0.002	
BT	1.04 (0.49–2.20)	0.927	
ADT	2.58 (1.64–4.05)	< 0.001	
Active surveillance/watchful-waiting	1.13 (0.57–2.25)	0.718	
Time since diagnosis, years			0.007
2–5	1.00	–	
5–10	0.85 (0.65–1.12)	0.251	
≥ 10	1.44 (1.06–1.95)	0.020	

^a^Unknown group was excluded if it was less than 5%

^b^From likelihood-ratio test

^c^Experienced both pre-diagnosis and post-diagnosis financial stress

^d^Early (localised disease: stage I/II and Gleason score 2–7 at diagnosis), late (locally advanced/advanced disease: III/IV and any Gleason score at diagnosis), unknown extent (other combinations of stage and Gleason score, or unknown stage or Gleason score)

^e^Hierarchical variable based on primary treatment(s) reported

*RoI* the Republic of Ireland; *NI* Northern Ireland; *RP* radical prostatectomy; *EBRT* external beam radiotherapy; *BT* brachytherapy; *ADT* androgen deprivation therapy

As regards other factors, living in NI (OR = 1.45, 95% CI 1.07–1.96), not working at diagnosis (1.62, 1.23–2.12), having comorbidities (1.76, 1.39–2.24), having late disease (1.61, 1.20–2.1), having received EBRT (1.57, 1.17–2.09) or ADT (2.58, 1.64–4.05) and having cancer diagnosed ≥ 10 years before the survey (1.44, 1.06–1.95) were significantly associated with a higher risk of CRF. Those who were 60–69 years of age at diagnosis (0.68, 0.51–0.92) and had at least tertiary level education (0.63, 0.46–0.86) had lower risk of CRF (Table 3).

## Discussion

Our results suggest that both clinically important CRF and financial stress are common problems for PCa survivors, even those who are several years from diagnosis. When present, CRF was strongly associated with financial stress, with a suggestion of a dose-response relationship such that survivors who experienced both pre-diagnosis and post-diagnosis financial stress had almost 5 times higher risk of CRF than those without financial stress; this relationship was not explained by socio-demographic and clinical differences between those with and without financial stress.

### Prevalence of financial hardship

There is growing recognition of the issue of financial stress, hardship or toxicity, among cancer survivors, including those with PCa [22, 39, 40]. In the current study, almost one quarter of PCa survivors (23.9%) reported post-diagnosis financial stress as a result of the cancer. It is difficult to compare prevalence across studies as investigators have rarely used the same questions/instruments to assess hardship. Moreover, the wider context within which the studies have been undertaken varies substantially. Both healthcare and social welfare systems, and employment legislation, will influence the likelihood that an individual experiences material financial stress due to cancer, since these affect, for example, the extent to which patients may incur cancer-related out-of-pocket costs and their entitlement to compensation for lost income when absent from work due to cancer. The prevalence of cancer-related financial stress in the current study is lower than that in previous studies of survivors of other types of cancer in RoI which used the same question (colorectal cancer, 41%; head and neck cancer, 51%; breast cancer, 51%) [23, 37, 41]. This is likely to be a function of, on average, the higher socio-economic status of men diagnosed with PCa [42]—which may provide, for many, a buffer against financial stress due to cancer—and the fact that this study population largely comprised longer-term survivors. Cancer-related financial hardship may lessen over time as survivors return to work and their income is restored and they ceased to have as many cancer-related out-of-pocket expenses (although empirical data is lacking on this) or they retire as they age.

### Financial hardship and CRF

The primary focus of the current study was not financial hardship per se but rather its association with clinically significant CRF. While there is a growing awareness of the link between cancer-related financial hardship and HRQoL among survivors [23, 28, 43], there has been much less investigation of associations between hardship and cancer-related symptoms. A recent systematic review reported that six studies have found relationships between financial toxicity and depression and three have reported relationships with anxiety [39]. In terms of physical symptoms, a large US study of colorectal and lung cancer found that financial toxicity was an independent predictor of cancer-related pain at 4 and 12 months post-diagnosis [44], while a study in France of a mixed group of cancers found a significant relationship between financial toxicity and overall physical symptom burden [45] Our results extend these by suggesting a relationship between financial hardship and CRF and—moreover—that those with financial stress before diagnosis who then experience cancer-related financial stress have substantially increased risk of CRF. CRF has a profound impact on PCa survivors’ lives, and is a major determinant of QoL [46, 47]. Studies on cancer-related symptoms, such as this one, are starting to suggest pathways by which financial hardship may lead to reduced QoL among survivors.

In terms of explanations for our findings, experiencing cancer-related symptoms (including fatigue and pain) can hamper cancer survivors’ ability to work, and increase their risk of unemployment and early retirement [48–51]. This suggests that the effects of CRF on work participation may, at least in part, explain our findings. Being at work is important for maintaining income in cancer survivors, and those who are absent from work due to cancer, or have to retire early, are likely to have a drop in income. Previous research in Ireland shows that many survivors (including those with PCa) do not receive financial compensation for time away from work due to the cancer and experience a drop in income post-diagnosis [52, 53]. A drop in income—due to an inability to work as a result of cancer-related symptoms (such as CRF)—could lead to post-diagnosis financial stress. Having noted this, only around half of survivors in the current study were working at diagnosis and less than three quarters of those working took time off. So it seems unlikely that this can entirely explain our findings.

Evidence suggests that physical activity and/or psychological interventions are effective in alleviating CRF [54, 55] and guidelines recommend somewhat different approaches depending on whether the individual is on active treatment, is post-treatment or at end-of-life. It is possible that survivors who experience financial stress may have reduced access to such interventions, either directly (i.e. for those interventions that require survivors to pay for them) or indirectly (because of reduced access to the healthcare services that would provide a route to the interventions). For example, pharmacological intervention would be likely to be prescribed by GPs and, in the RoI, most individuals make co-payments to be seen by a GP and pay the full cost of prescribed medications; those experiencing financial stress may struggle to make these payments. This provides another possible explanation for our findings.

In the general population, material hardship—at both the individual and household level—is associated with worse self-rated health [56, 57]. Moreover, self-rated health and fatigue are strongly inversely related [58]. Although CRF is different from fatigue that is not associated with cancer, it is noteworthy that our observations about CRF among PCa survivors broadly reflect patterns in the general population.

In terms of how financial distress may lead to CRF, it is established that psychological distress is a predictor of CRF in PCa survivors [59]. There are inter-relationships between the economic impact of cancer on the individual and the emotional distress they experience [60, 61]. Thus, a possible pathway may involve financial stress influencing emotional distress and that then influencing CRF.

Current research on the biological mechanisms of CRF suggests an important role for proinflammatory cytokines, which contribute to several relevant pathways (e.g. neuroendocrine-immune signalling) [62, 63]. Factors such as financial stressors, depression and elevated distress can influence inflammation activity and—potentially—contribute to CRF [63]. This is thus a possible underlying mechanism for the associations observed here. Of course, this is speculative and empirical work measuring inflammation activity in survivors with and without financial stress would be valuable.

### Other risk factors for CRF

In terms of other risk factors for CRF, comparisons with other studies are complicated by the fact that these generally included people with other types of cancer or selected groups of PCa survivors; treatment is a key determinant of CRF [9] and, of course, treatment varies by cancer site and extent of disease. A further complication is that studies have used different instruments to assess CRF. Having said that, there are some consistencies between our findings and others. For example, previous studies, in breast, colorectal and prostate cancer, have reported increased risk of CRF in survivors who have comorbidities [14, 64, 65]. However, findings regarding educational level and CRF are more inconsistent [14, 64, 66, 67].

In the current study, very-long-term survivors (≥ 10 years from diagnosis) had a 44% increased risk of CRF. These survivors may be more likely to have been discharged from hospital-based follow-up and therefore not be in regular contact with health professionals who might be able to help manage CRF. This group of survivors were also older at the time of questionnaire completion. Previous work has shown that older survivors report higher levels of fatigue, and that both cancer-related and age-related factors contribute to this [68].

### Limitations

There are several limitations in our study. Compared to the entire population of PCa survivors on the island of Ireland, among respondents, older men (70 + at diagnosis) were under-represented and shorter-term survivors (2–4.99 years from diagnosis) were over-represented [33]. In the current analysis, the study population was younger at diagnosis, more often from RoI and more often had early-stage disease than respondents who did not provide complete data on CRF and financial stress. It is therefore possible that the overall estimates of the prevalence of CRF and financial hardship were biased. For example, since financial stress was less commonly reported among younger survivors, we may have overestimated the overall prevalence of financial stress. In contrast, because CRF was more common among older survivors, the overall prevalence of CRF may be underestimated.

Both financial stress and clinically important CRF were based on self-report. The former was assessed using questions that have previously been found to have convergent validity with objective measures of financial burden in cancer survivors in Ireland [28, 41]. However, we did not have data on individual financial stressors or measurements of financial stress over time. Moreover, it is likely that there is some inaccuracy in participants’ recall of whether they experienced financial stress; this will have resulted in misclassification. CRF was measured using a validated HRQoL instrument and a cutoff that has been validated for clinically important fatigue [35, 36]. However, the EORTC QLQ-C30 fatigue subscale includes only three questions. Other more extensive tools are available which, for example, distinguish different manifestations of CRF (e.g. physical, emotional, cognitive) and/or assess the extent to which CRF interferes with daily life [69, 70]. The possibility cannot be excluded that our results would have differed had we used one of these instruments. Research investigating relationships between the financial impact of PCa and different aspects of CRF would be valuable.

As with any report of an association between two variables, it is possible that the results may be affected by uncontrolled confounding. In this case, the lack of previous investigations of financial stress and CRF make it difficult to speculate as to what uncontrolled confounders could explain, in full or in part, the results. We lacked information on some potential influences on CRF, such as chemotherapy receipt [46] at time of survey completion. While only two participants had had chemotherapy as initial treatment for their prostate cancer, it is possible that some were undergoing chemotherapy at the time of survey completion because their disease had progressed. If chemotherapy receipt was more common in those reporting financial stress, this could have impacted our findings. Finally, as the distributions of potential determinants of CRF vary across different populations, our findings may not be generalised to other populations.

### Implications

Our findings imply that screening for financial stress may help identify survivors at higher risk of CRF during and after treatment. In the USA, screening for financial toxicity among cancer patients has been advocated [71] and a recently developed measure of financial toxicity [72] might be useful in this regard. Such screening would be valuable on its own account as it would help identify those who might benefit from financial support and advice, but it is also possible that interventions to prevent or ameliorate CRF may be more effective if either they are targeted towards those experiencing (or at risk of) financial distress or measures are first taken to help survivors address financial stress.

In an ideal world, survivors would be fully protected from, or compensated for, cancer-related financial stress, but this may be unrealistic in lean economic times. Measures that may be more feasible—but still helpful—include financial and/or welfare advice services, especially if these have specific cancer expertise, and support funds which assist with treatment-related travel or accommodation costs [73–76]. Other useful initiatives include training and resources for employers to enable them to support survivors to return to work [77].

As regards research, there is a need for longitudinal studies of cancer-related financial stress, its determinants and its relationship with CRF (and other cancer-related symptoms), and inter-relationships with emotional distress and HRQoL among well-defined PCa survivor populations. Such studies are needed in settings with different healthcare and social welfare provisions, since findings from one context may not easily generalise to another.

## Conclusions

In conclusion, our findings show that both clinically important CRF and financial stress are common problems for PCa survivors, even those who are several years from diagnosis. Moreover, cumulative financial stress exposure may be an independent risk factor for CRF among PCa survivors. There may be benefits in targeting interventions for reducing CRF towards survivors with financial stress, or developing strategies to reduce financial stress among survivors.

## Electronic supplementary material

ESM 1 (DOCX 27 kb).
