# Supplementary material for: Cumulative financial stress as a potential risk factor for cancer-related fatigue among prostate cancer survivors
Source: J Cancer Surviv. 2020 Aug 1;15(1):1–13. doi: 10.1007/s11764-020-00906-7 (PMC7822770; doi:10.1007/s11764-020-00906-7)
Supplement: Supplementary file 1 — (DOCX 27 kb). [file 11764_2020_906_MOESM1_ESM.docx]

**Supplementary Table 1. Characteristics of prostate cancer survivors included in the analysis^a^ by levels of financial stress exposure**

|  | **Financial stress** | | | | **p^b^** |
| --- | --- | --- | --- | --- | --- |
|  | **No**  **(n=1603)** | **Pre-diagnosis only (n=268)** | **Post-diagnosis only (n=317)** | **Cumulative^c^ (n=270)** |  |
|  | **n (%)** | **n (%)** | **n (%)** | **n (%)** |  |
| **Age at diagnosis, years** |  |  |  |  |  |
| ≤59 | 399 (56.2) | 80 (11.3) | 136 (19.1) | 95 (13.4) | <0.001 |
| 60-69 | 823 (68.6) | 120 (10.0) | 134 (11.2) | 122 (10.2) |  |
| ≥ 70 | 381 (69.4) | 68 (12.4) | 47 (8.6) | 53 (9.6) |  |
| **Jurisdiction** |  |  |  |  |  |
| RoI | 1283 (64.1) | 241 (12.0) | 249 (12.4) | 229 (11.5) | <0.001 |
| NI | 320 (70.2) | 68 (5.9) | 68 (14.9) | 41 (9.0) |  |
| **Marital status at diagnosis** |  |  |  |  |  |
| Married/ living with a partner | 1345 (65.7) | 219 (10.7) | 264 (12.9) | 219 (10.7) | 0.853 |
| Other | 250 (63.5) | 45 (11.4) | 53 (13.4) | 46 (11.7) |  |
| Not reported | 8 (47.1) | 4 (23.5) | 0 | 5 (29.4) |  |
| **Live alone at diagnosis** |  |  |  |  |  |
| No | 1403 (65.3) | 237 (11.0) | 280 (13.0) | 227 (10.6) | 0.144 |
| Yes | 183 (63.3) | 26 (9.0) | 37 (12.8) | 43 (14.9) |  |
| Not reported | 17 (77.3) | 5 (22.7) | 0 | 0 |  |
| **Highest level of education at diagnosis** |  |  |  |  |  |
| Primary | 448 (56.7) | 111 (14.0) | 98 (12.4) | 134 (16.9) | <0.001 |
| Secondary | 616 (67.4) | 93 (10.2) | 113 (12.3) | 92 (10.1) |  |
| ≥Tertiary | 495 (73.3) | 51 (7.6) | 99 (14.7) | 30 (4.4) |  |
| Not reported | 44 (56.4) | 13 (16.7) | 7 (9.0) | 14 (17.9) |  |
| **Employment status, immediately before diagnosis** |  |  |  |  |  |
| Working | 835 (63.2) | 126 (9.5) | 232 (17.5) | 129 (9.8) | <0.001 |
| Not working | 717 (69.0) | 127 (12.2) | 78 (7.5) | 117 (11.3) |  |
| Not reported | 51 (52.6) | 15 (15.5) | 7 (7.2) | 24 (24.7) |  |
| **Comorbidities at diagnosis** |  |  |  |  |  |
| No | 786 (68.5) | 116 (10.1) | 138 (12.0) | 108 (9.4) | 0.012 |
| Yes | 817 (62.4) | 152 (11.6) | 179 (13.7) | 162 (12.3) |  |
| **Extent of disease at diagnosis^d^** |  |  |  |  |  |
| Early | 874 (67.8) | 140 (10.8) | 156 (12.1) | 120 (9.3) | 0.010 |
| Late | 282 (61.6) | 43 (9.4) | 79 (17.2) | 54 (11.8) |  |
| Unknown | 447 (63.0) | 85 (12.0) | 82 (11.5) | 96 (13.5) |  |
| **Treatment^e^** |  |  |  |  |  |
| RP | 501 (62.5) | 80 (10.0) | 132 (16.4) | 89 (11.1) | <0.001 |
| EBRT | 789 (64.7) | 135 (11.1) | 146 (12.0) | 149 (12.2) |  |
| BT | 84 (77.1) | 13 (11.9) | 7 (6.4) | 5 (4.6) |  |
| ADT | 117 (66.1) | 18 (10.2) | 26 (14.7) | 16 (9.0) |  |
| Active surveillance/watchful-waiting | 79 (76.0) | 15 (14.4) | 1 (1.0) | 9 (8.6) |  |
| Other | 33 (70.2) | 7 (14.9) | 5 (10.6) | 2 (4.3) |  |
| **Time since diagnosis, years** |  |  |  |  |  |
| 2-5 | 750 (63.9) | 136 (11.6.8) | 154 (13.1) | 134 (11.4) | 0.675 |
| 5-10 | 528 (67.2) | 74 (9.4) | 103 (13.1) | 81 (10.3) |  |
| ≥ 10 | 325 (65.3) | 58 (11.6) | 60 (12.1) | 55 (11.0) |  |

^a^ N=2458

^b^ from χ^2^ test comparing socio-demographic and clinical characteristics across financial stress exposure levels

^c^ experienced both pre-diagnosis and post-diagnosis financial stress

^d^ early (localised disease: stage I/II and Gleason score 2-7 at diagnosis), late (locally advanced/advanced disease: III/IV and any Gleason score at diagnosis), unknown extent (other combinations of stage and Gleason score, or unknown stage or Gleason score)

^e^ primary treatment(s): a hierarchical variable defined as i) RP at any time following diagnosis (with/without other treatments); ii) EBRT with/without concurrent ADT; iii) BT without previous RP or EBRT; iv) ADT alone without RP, EBRT or BT; v) active surveillance/watchful-waiting; and vi) other (which includes chemotherapy (2 participants) and unknown treatment (45 participants))

RoI the Republic of Ireland; NI Northern Ireland; RP radical prostatectomy; EBRT external beam radiotherapy; BT brachytherapy; ADT androgen deprivation therapy
